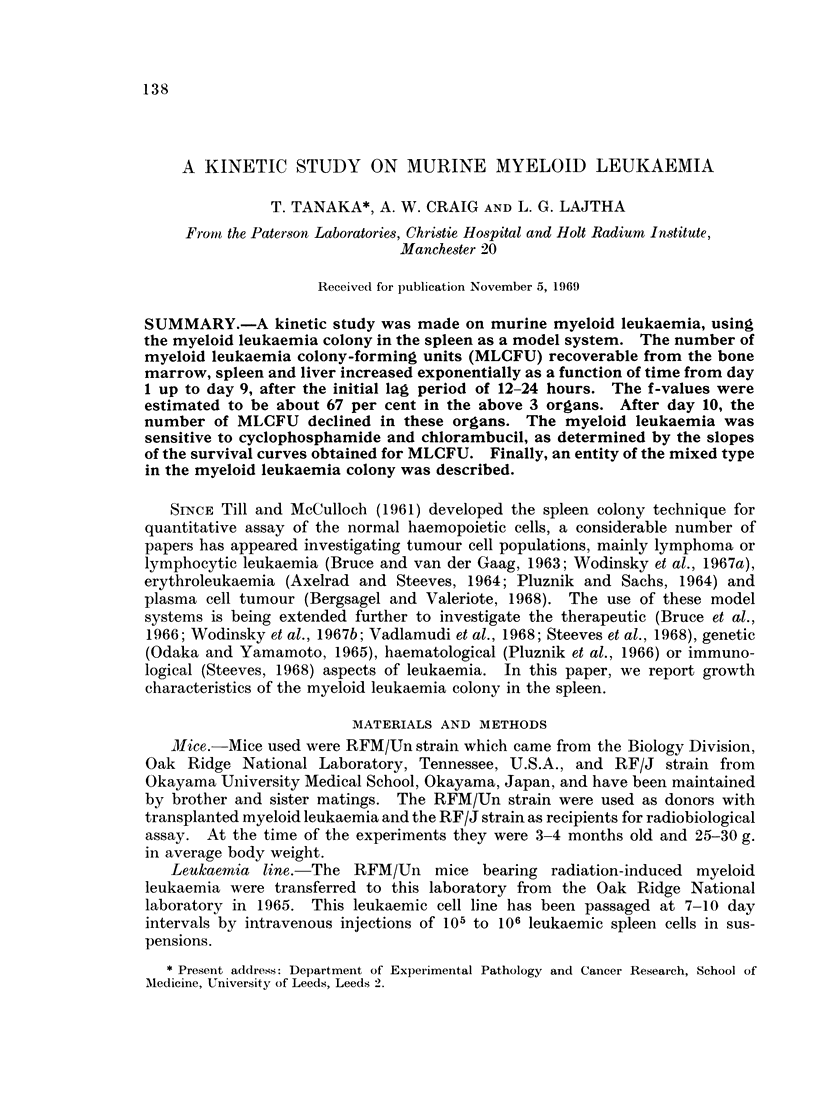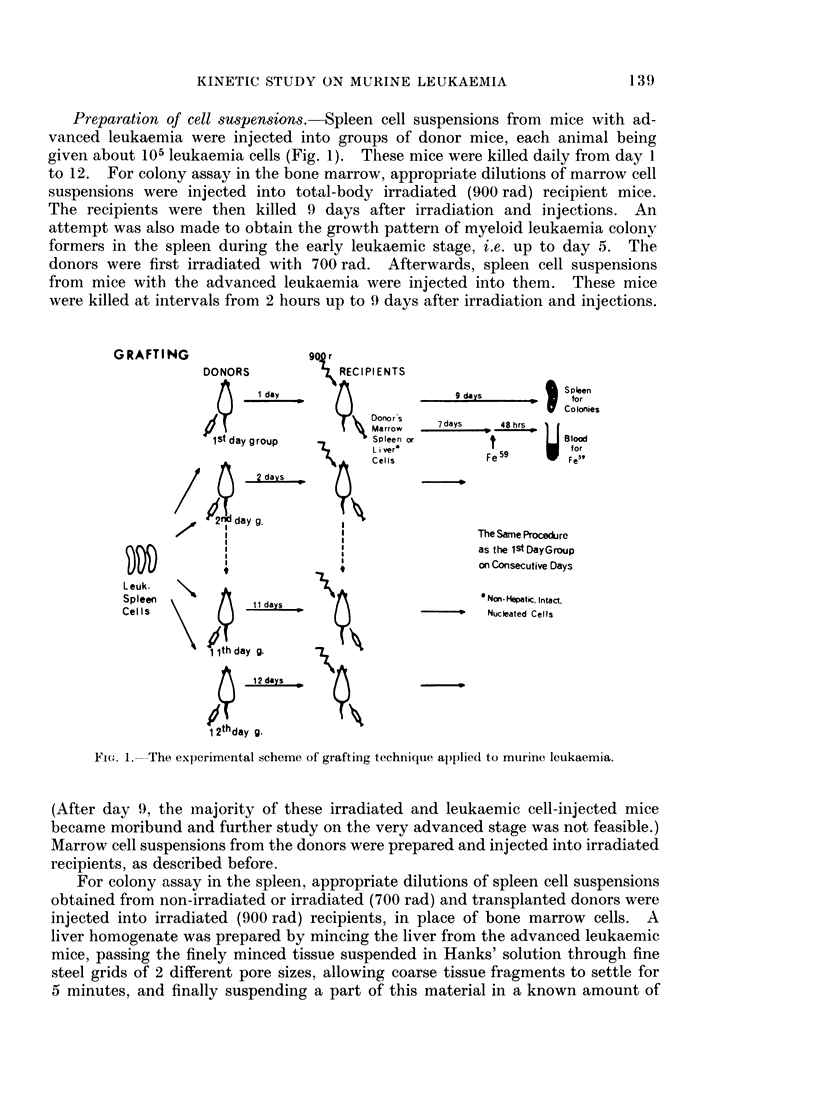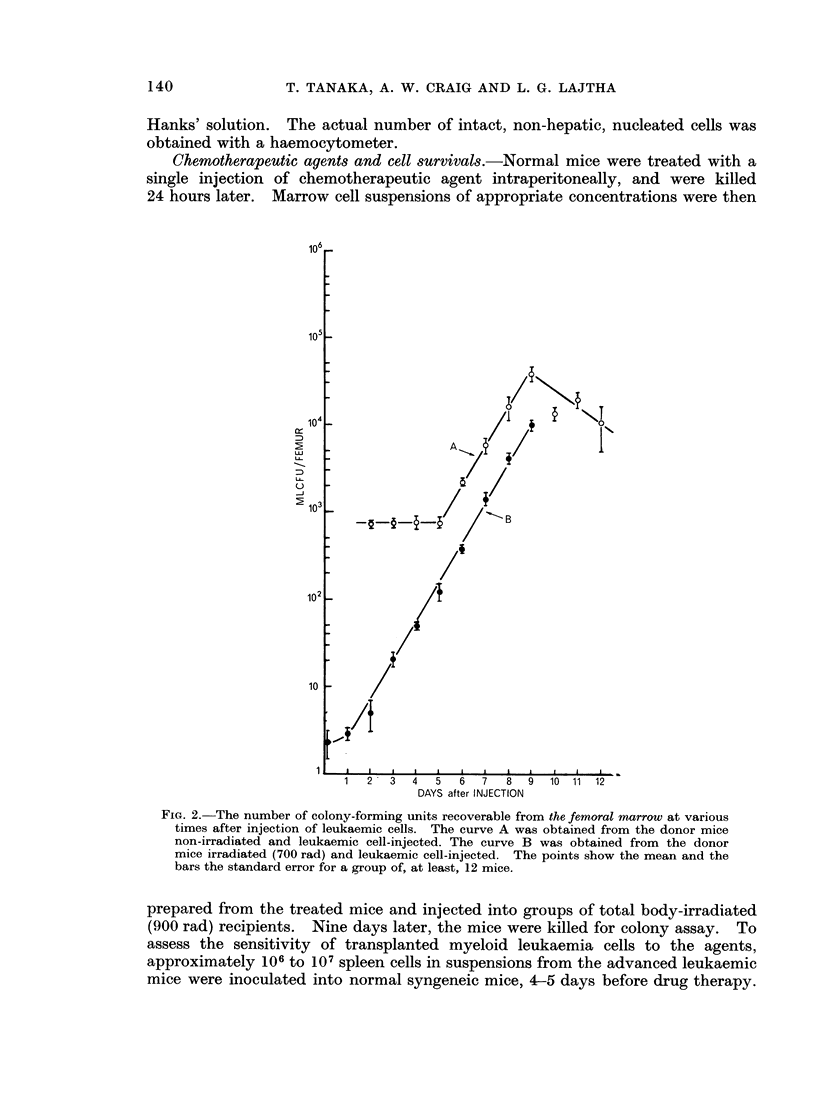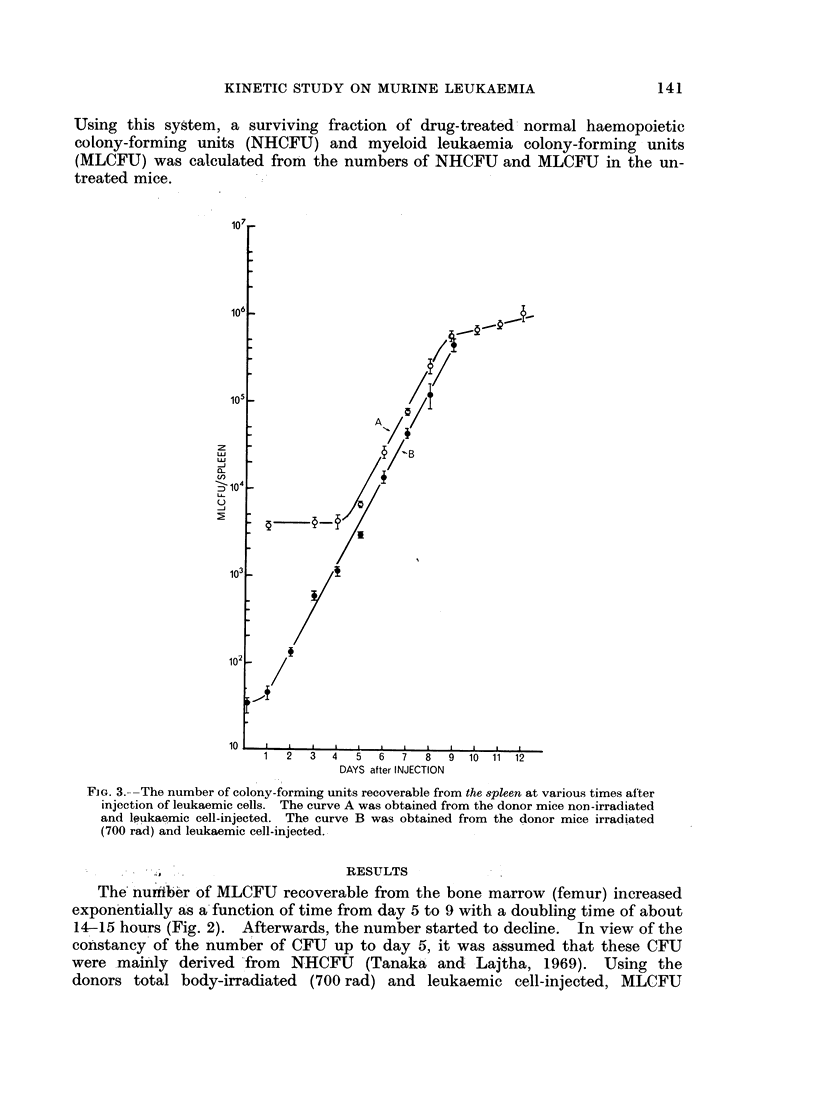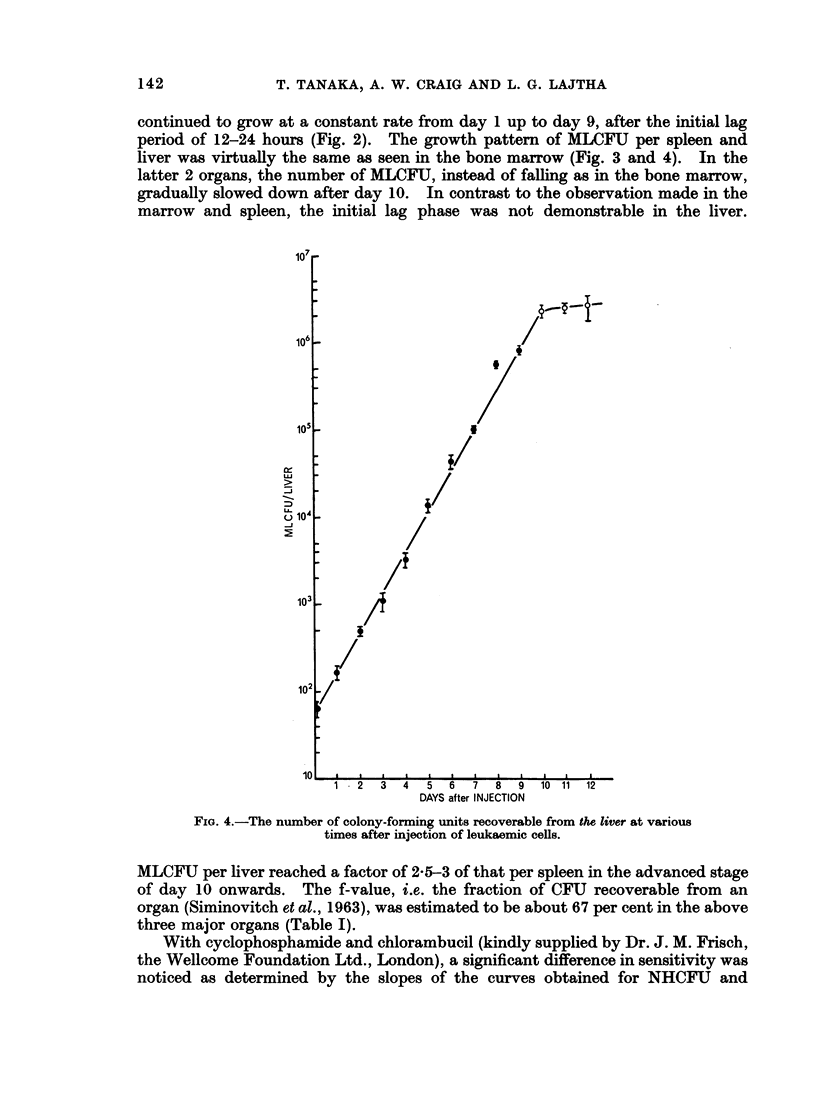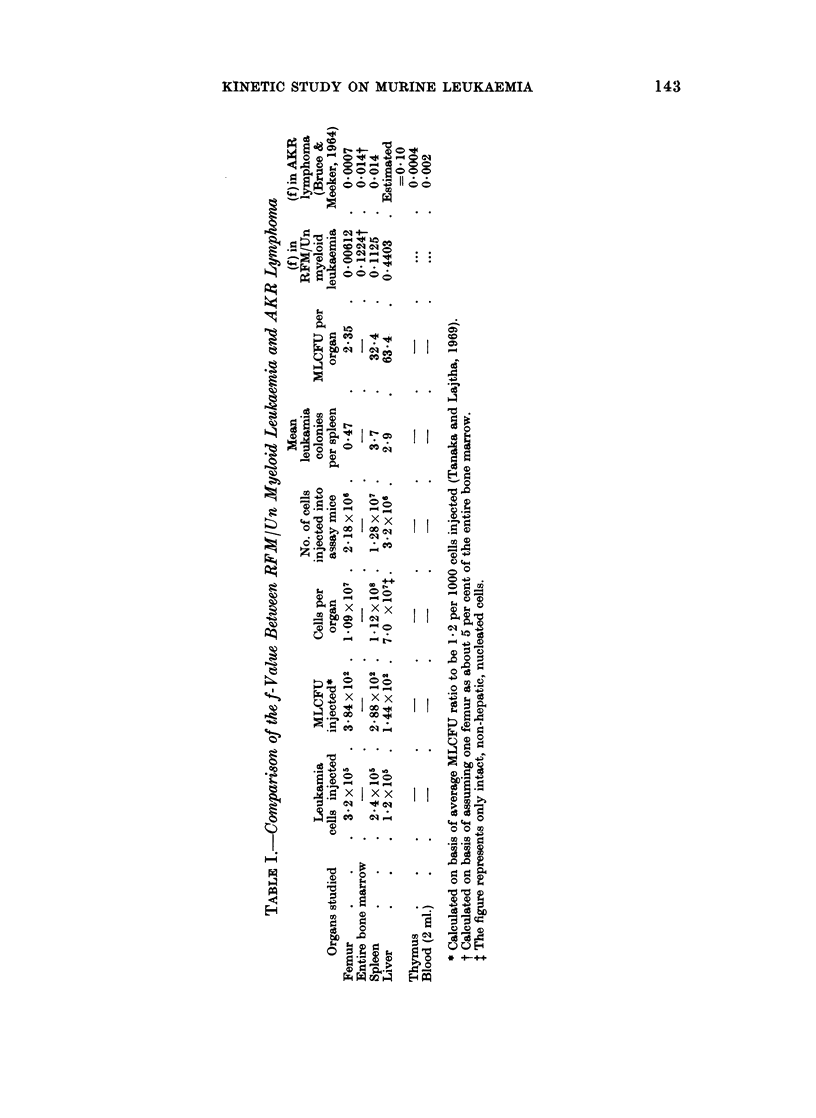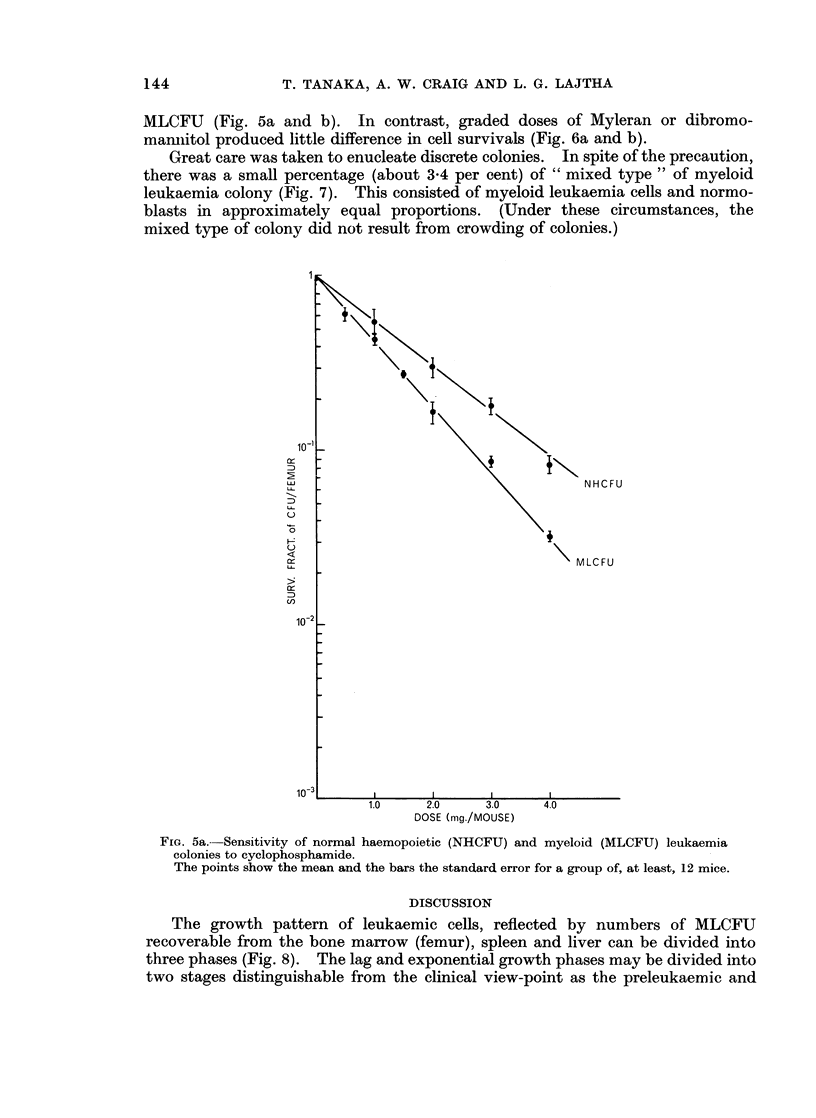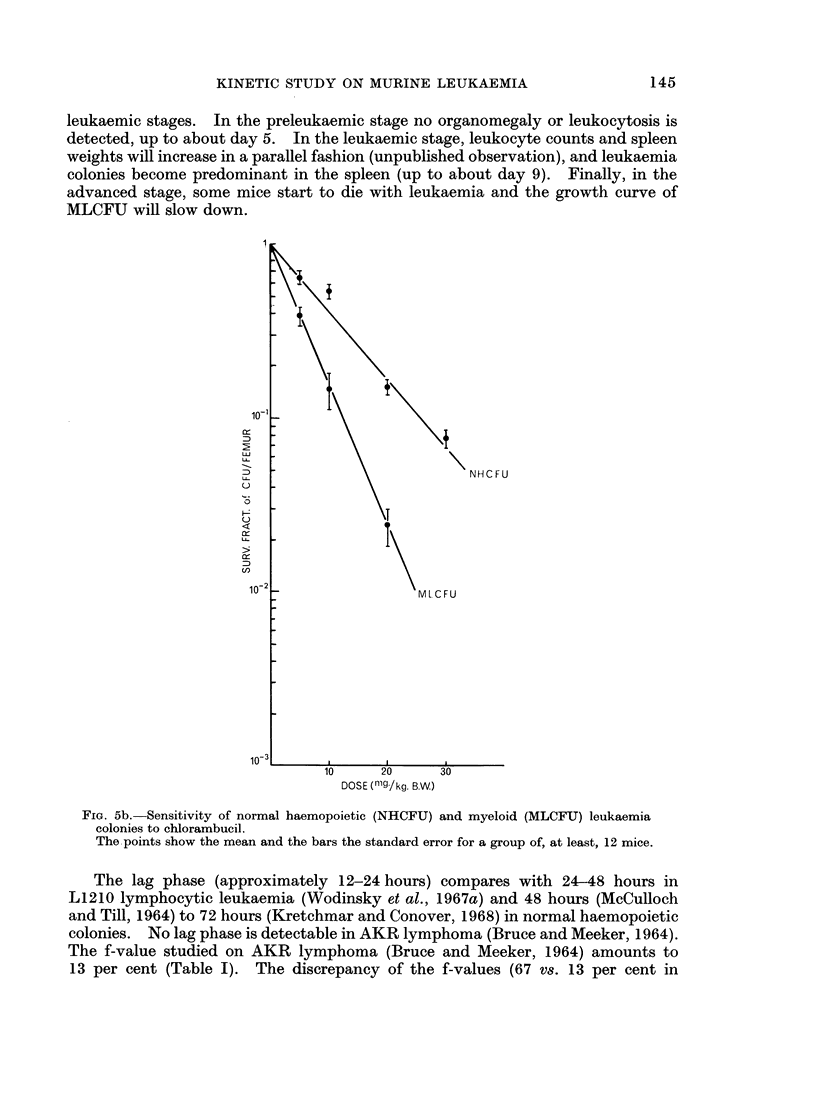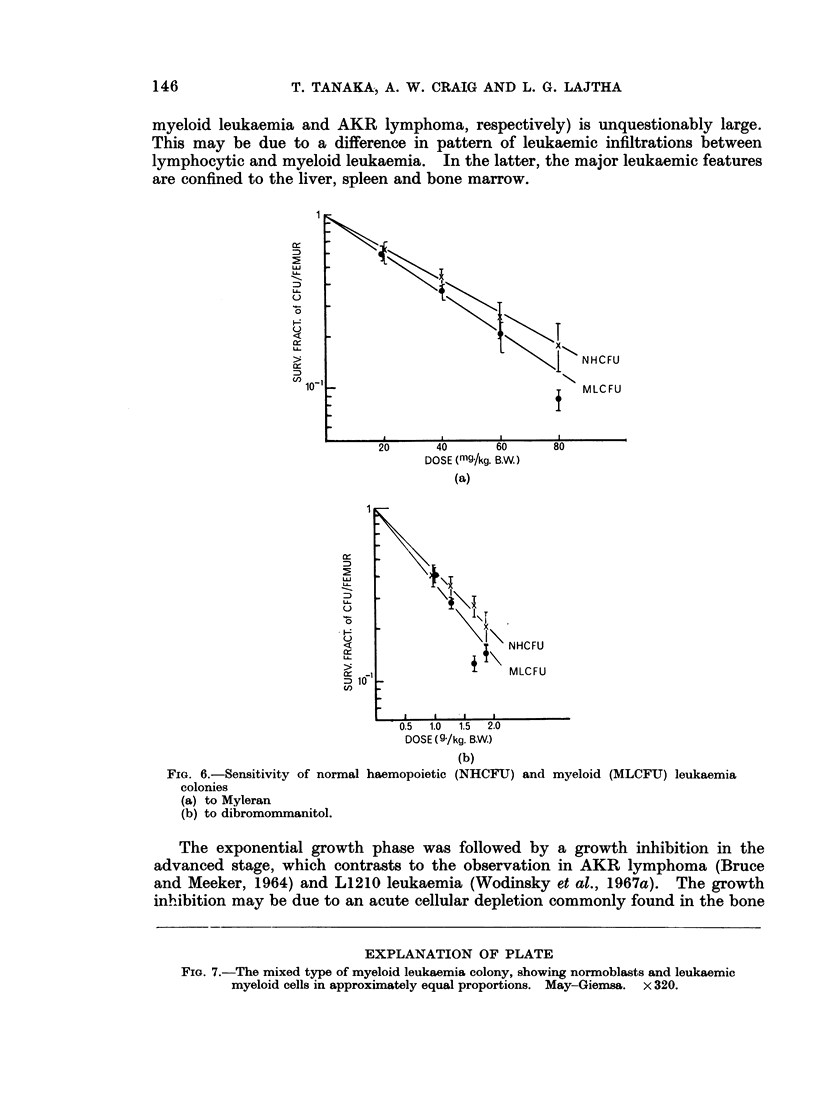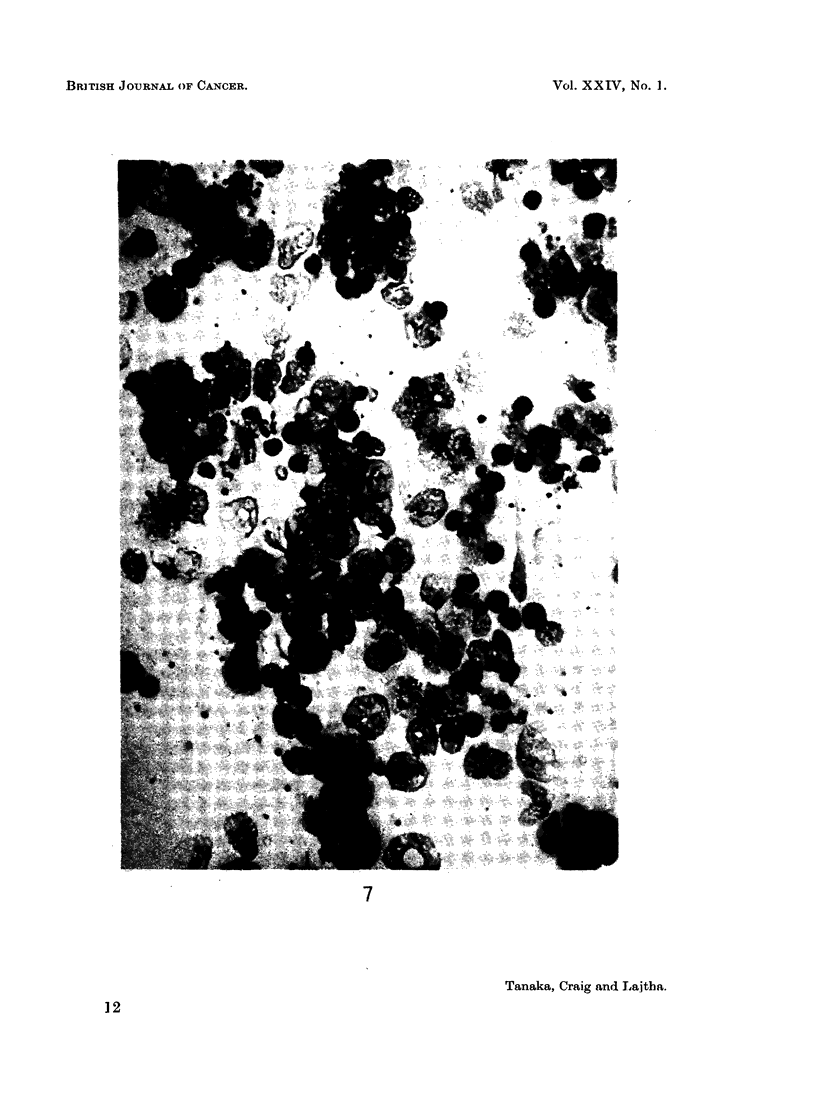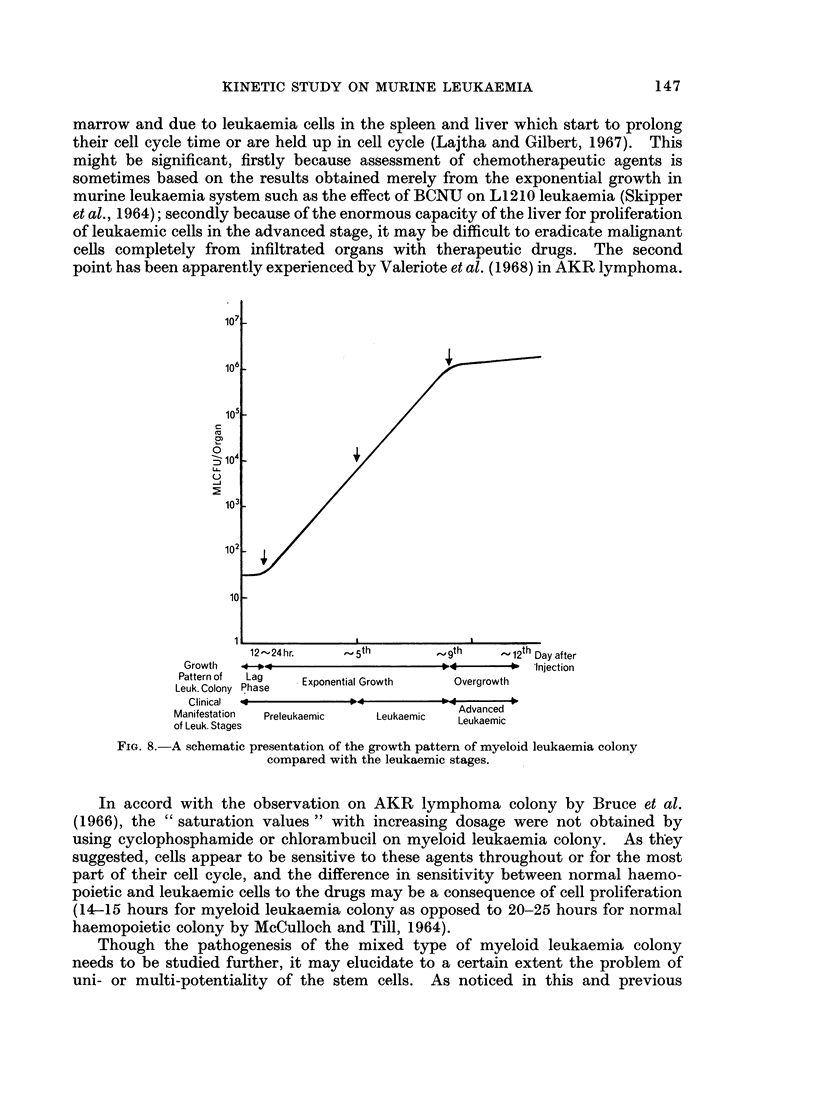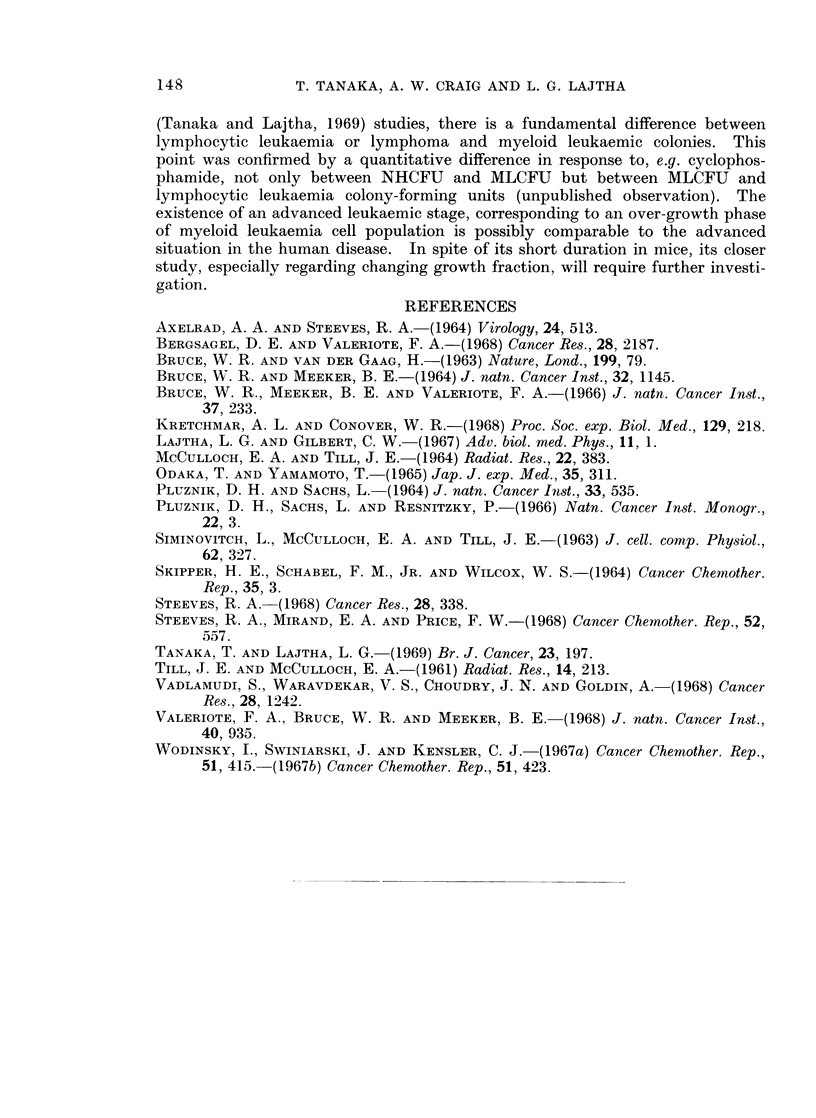# A Kinetic Study On Murine Myeloid Leukaemia

**DOI:** 10.1038/bjc.1970.17

**Published:** 1970-03

**Authors:** T. Tanaka, A. W. Craig, L. G. Lajtha

## Abstract

**Images:**


### A KINETIC STUDY ON MURINE MYELOID LEUKAEMIA

T. TANAKA*, A. W. CRAIG AND L. G. LAJTHA

*From the Paterson Laboratories, Christie Hospital and Holt Radium Institute, Manchester 20*

Received for publication November 5, 1969

**SUMMARY.—A kinetic study was made on murine myeloid leukaemia, using the myeloid leukaemia colony in the spleen as a model system. The number of myeloid leukaemia colony-forming units (MLCFU) recoverable from the bone marrow, spleen and liver increased exponentially as a function of time from day 1 up to day 9, after the initial lag period of 12–24 hours. The f-values were estimated to be about 67 per cent in the above 3 organs. After day 10, the number of MLCFU declined in these organs. The myeloid leukaemia was sensitive to cyclophosphamide and chlorambucil, as determined by the slopes of the survival curves obtained for MLCFU. Finally, an entity of the mixed type in the myeloid leukaemia colony was described.**

SINCE Till and McCulloch (1961) developed the spleen colony technique for quantitative assay of the normal haemopoietic cells, a considerable number of papers has appeared investigating tumour cell populations, mainly lymphoma or lymphocytic leukaemia (Bruce and van der Gaag, 1963; Wodinsky *et al.*, 1967*a*), erythroleukaemia (Axelrad and Steeves, 1964; Pluznik and Sachs, 1964) and plasma cell tumour (Bergsagel and Valeriote, 1968). The use of these model systems is being extended further to investigate the therapeutic (Bruce *et al.*, 1966; Wodinsky *et al.*, 1967*b*; Vadlamudi *et al.*, 1968; Steeves *et al.*, 1968), genetic (Odaka and Yamamoto, 1965), haematological (Pluznik *et al.*, 1966) or immunological (Steeves, 1968) aspects of leukaemia. In this paper, we report growth characteristics of the myeloid leukaemia colony in the spleen.

### MATERIALS AND METHODS

*Mice.*—Mice used were RFM/Un strain which came from the Biology Division, Oak Ridge National Laboratory, Tennessee, U.S.A., and RF/J strain from Okayama University Medical School, Okayama, Japan, and have been maintained by brother and sister matings. The RFM/Un strain were used as donors with transplanted myeloid leukaemia and the RF/J strain as recipients for radiobiological assay. At the time of the experiments they were 3–4 months old and 25–30 g. in average body weight.

*Leukaemia line.*—The RFM/Un mice bearing radiation-induced myeloid leukaemia were transferred to this laboratory from the Oak Ridge National laboratory in 1965. This leukaemic cell line has been passaged at 7–10 day intervals by intravenous injections of $10^5$ to $10^6$ leukaemic spleen cells in suspensions.

* Present address: Department of Experimental Pathology and Cancer Research, School of Medicine, University of Leeds, Leeds 2.

*Preparation of cell suspensions.*—Spleen cell suspensions from mice with advanced leukaemia were injected into groups of donor mice, each animal being given about $10^5$ leukaemia cells (Fig. 1). These mice were killed daily from day 1 to 12. For colony assay in the bone marrow, appropriate dilutions of marrow cell suspensions were injected into total-body irradiated (900 rad) recipient mice. The recipients were then killed 9 days after irradiation and injections. An attempt was also made to obtain the growth pattern of myeloid leukaemia colony formers in the spleen during the early leukaemic stage, *i.e.* up to day 5. The donors were first irradiated with 700 rad. Afterwards, spleen cell suspensions from mice with the advanced leukaemia were injected into them. These mice were killed at intervals from 2 hours up to 9 days after irradiation and injections.

FIG. 1.—The experimental scheme of grafting technique applied to murine leukaemia.

(After day 9, the majority of these irradiated and leukaemic cell-injected mice became moribund and further study on the very advanced stage was not feasible.) Marrow cell suspensions from the donors were prepared and injected into irradiated recipients, as described before.

For colony assay in the spleen, appropriate dilutions of spleen cell suspensions obtained from non-irradiated or irradiated (700 rad) and transplanted donors were injected into irradiated (900 rad) recipients, in place of bone marrow cells. A liver homogenate was prepared by mincing the liver from the advanced leukaemic mice, passing the finely minced tissue suspended in Hanks' solution through fine steel grids of 2 different pore sizes, allowing coarse tissue fragments to settle for 5 minutes, and finally suspending a part of this material in a known amount of

Hanks' solution. The actual number of intact, non-hepatic, nucleated cells was obtained with a haemocytometer.

*Chemotherapeutic agents and cell survivals.*—Normal mice were treated with a single injection of chemotherapeutic agent intraperitoneally, and were killed 24 hours later. Marrow cell suspensions of appropriate concentrations were then

FIG. 2.—The number of colony-forming units recoverable from *the femoral marrow* at various times after injection of leukaemic cells. The curve A was obtained from the donor mice non-irradiated and leukaemic cell-injected. The curve B was obtained from the donor mice irradiated (700 rad) and leukaemic cell-injected. The points show the mean and the bars the standard error for a group of, at least, 12 mice.

prepared from the treated mice and injected into groups of total body-irradiated (900 rad) recipients. Nine days later, the mice were killed for colony assay. To assess the sensitivity of transplanted myeloid leukaemia cells to the agents, approximately $10^6$ to $10^7$ spleen cells in suspensions from the advanced leukaemic mice were inoculated into normal syngeneic mice, 4–5 days before drug therapy.

Using this system, a surviving fraction of drug-treated normal haemopoietic colony-forming units (NHCFU) and myeloid leukaemia colony-forming units (MLCFU) was calculated from the numbers of NHCFU and MLCFU in the untreated mice.

FIG. 3.—The number of colony-forming units recoverable from *the spleen* at various times after injection of leukaemic cells. The curve A was obtained from the donor mice non-irradiated and leukaemic cell-injected. The curve B was obtained from the donor mice irradiated (700 rad) and leukaemic cell-injected.

### RESULTS

The number of MLCFU recoverable from the bone marrow (femur) increased exponentially as a function of time from day 5 to 9 with a doubling time of about 14–15 hours (Fig. 2). Afterwards, the number started to decline. In view of the constancy of the number of CFU up to day 5, it was assumed that these CFU were mainly derived from NHCFU (Tanaka and Lajtha, 1969). Using the donors total body-irradiated (700 rad) and leukaemic cell-injected, MLCFU

continued to grow at a constant rate from day 1 up to day 9, after the initial lag period of 12–24 hours (Fig. 2). The growth pattern of MLCFU per spleen and liver was virtually the same as seen in the bone marrow (Fig. 3 and 4). In the latter 2 organs, the number of MLCFU, instead of falling as in the bone marrow, gradually slowed down after day 10. In contrast to the observation made in the marrow and spleen, the initial lag phase was not demonstrable in the liver.

FIG. 4.—The number of colony-forming units recoverable from *the liver* at various times after injection of leukaemic cells.

MLCFU per liver reached a factor of 2·5–3 of that per spleen in the advanced stage of day 10 onwards. The f-value, *i.e.* the fraction of CFU recoverable from an organ (Siminovitch *et al.*, 1963), was estimated to be about 67 per cent in the above three major organs (Table I).

With cyclophosphamide and chlorambucil (kindly supplied by Dr. J. M. Frisch, the Wellcome Foundation Ltd., London), a significant difference in sensitivity was noticed as determined by the slopes of the curves obtained for NHCFU and

TABLE I.—*Comparison of the f-Value Between RFM/Un Myeloid Leukaemia and AKR Lymphoma*

| Organs studied | Leukamia cells injected | MLCFU injected* | Cells per organ | No. of cells injected into assay mice | Mean leukamia colonies per spleen | MLCFU per organ | (f) in RFM/Un myeloid leukaemia | (f) in AKR lymphoma (Bruce & Meeker, 1964) |
|---|---|---|---|---|---|---|---|---|
| Femur | $3\cdot2\times10^5$ | $3\cdot84\times10^2$ | $1\cdot09\times10^7$ | $2\cdot18\times10^6$ | 0·47 | 2·35 | 0·00612 | 0·0007 |
| Entire bone marrow | — | — | — | — | — | — | 0·1224† | 0·014† |
| Spleen | $2\cdot4\times10^5$ | $2\cdot88\times10^2$ | $1\cdot12\times10^8$ | $1\cdot28\times10^7$ | 3·7 | 32·4 | 0·1125 | 0·014 |
| Liver | $1\cdot2\times10^5$ | $1\cdot44\times10^2$ | $7\cdot0\times10^7$‡ | $3\cdot2\times10^6$ | 2·9 | 63·4 | 0·4403 | Estimated =0·10 |
| Thymus | — | — | — | — | — | — | ... | 0·0004 |
| Blood (2 ml.) | — | — | — | — | — | — | ... | 0·002 |

* Calculated on basis of average MLCFU ratio to be 1·2 per 1000 cells injected (Tanaka and Lajtha, 1969).

† Calculated on basis of assuming one femur as about 5 per cent of the entire bone marrow.

‡ The figure represents only intact, non-hepatic, nucleated cells.

MLCFU (Fig. 5a and b). In contrast, graded doses of Myleran or dibromomannitol produced little difference in cell survivals (Fig. 6a and b).

Great care was taken to enucleate discrete colonies. In spite of the precaution, there was a small percentage (about 3·4 per cent) of " mixed type " of myeloid leukaemia colony (Fig. 7). This consisted of myeloid leukaemia cells and normoblasts in approximately equal proportions. (Under these circumstances, the mixed type of colony did not result from crowding of colonies.)

FIG. 5a.—Sensitivity of normal haemopoietic (NHCFU) and myeloid (MLCFU) leukaemia colonies to cyclophosphamide.

The points show the mean and the bars the standard error for a group of, at least, 12 mice.

### DISCUSSION

The growth pattern of leukaemic cells, reflected by numbers of MLCFU recoverable from the bone marrow (femur), spleen and liver can be divided into three phases (Fig. 8). The lag and exponential growth phases may be divided into two stages distinguishable from the clinical view-point as the preleukaemic and leukaemic stages. In the preleukaemic stage no organomegaly or leukocytosis is detected, up to about day 5. In the leukaemic stage, leukocyte counts and spleen weights will increase in a parallel fashion (unpublished observation), and leukaemia colonies become predominant in the spleen (up to about day 9). Finally, in the advanced stage, some mice start to die with leukaemia and the growth curve of MLCFU will slow down.

FIG. 5b.—Sensitivity of normal haemopoietic (NHCFU) and myeloid (MLCFU) leukaemia colonies to chlorambucil.
The points show the mean and the bars the standard error for a group of, at least, 12 mice.

The lag phase (approximately 12–24 hours) compares with 24–48 hours in L1210 lymphocytic leukaemia (Wodinsky *et al.*, 1967*a*) and 48 hours (McCulloch and Till, 1964) to 72 hours (Kretchmar and Conover, 1968) in normal haemopoietic colonies. No lag phase is detectable in AKR lymphoma (Bruce and Meeker, 1964). The f-value studied on AKR lymphoma (Bruce and Meeker, 1964) amounts to 13 per cent (Table I). The discrepancy of the f-values (67 *vs.* 13 per cent in myeloid leukaemia and AKR lymphoma, respectively) is unquestionably large. This may be due to a difference in pattern of leukaemic infiltrations between lymphocytic and myeloid leukaemia. In the latter, the major leukaemic features are confined to the liver, spleen and bone marrow.

(a)

(b)

FIG. 6.—Sensitivity of normal haemopoietic (NHCFU) and myeloid (MLCFU) leukaemia colonies
(a) to Myleran
(b) to dibromommanitol.

The exponential growth phase was followed by a growth inhibition in the advanced stage, which contrasts to the observation in AKR lymphoma (Bruce and Meeker, 1964) and L1210 leukaemia (Wodinsky *et al.*, 1967*a*). The growth inhibition may be due to an acute cellular depletion commonly found in the bone

EXPLANATION OF PLATE

FIG. 7.—The mixed type of myeloid leukaemia colony, showing normoblasts and leukaemic myeloid cells in approximately equal proportions. May–Giemsa. ×320.

Tanaka, Craig and Lajtha.

marrow and due to leukaemia cells in the spleen and liver which start to prolong their cell cycle time or are held up in cell cycle (Lajtha and Gilbert, 1967). This might be significant, firstly because assessment of chemotherapeutic agents is sometimes based on the results obtained merely from the exponential growth in murine leukaemia system such as the effect of BCNU on L1210 leukaemia (Skipper *et al.*, 1964); secondly because of the enormous capacity of the liver for proliferation of leukaemic cells in the advanced stage, it may be difficult to eradicate malignant cells completely from infiltrated organs with therapeutic drugs. The second point has been apparently experienced by Valeriote *et al.* (1968) in AKR lymphoma.

FIG. 8.—A schematic presentation of the growth pattern of myeloid leukaemia colony compared with the leukaemic stages.

In accord with the observation on AKR lymphoma colony by Bruce *et al.* (1966), the " saturation values " with increasing dosage were not obtained by using cyclophosphamide or chlorambucil on myeloid leukaemia colony. As they suggested, cells appear to be sensitive to these agents throughout or for the most part of their cell cycle, and the difference in sensitivity between normal haemopoietic and leukaemic cells to the drugs may be a consequence of cell proliferation (14–15 hours for myeloid leukaemia colony as opposed to 20–25 hours for normal haemopoietic colony by McCulloch and Till, 1964).

Though the pathogenesis of the mixed type of myeloid leukaemia colony needs to be studied further, it may elucidate to a certain extent the problem of uni- or multi-potentiality of the stem cells. As noticed in this and previous

(Tanaka and Lajtha, 1969) studies, there is a fundamental difference between lymphocytic leukaemia or lymphoma and myeloid leukaemic colonies. This point was confirmed by a quantitative difference in response to, *e.g.* cyclophosphamide, not only between NHCFU and MLCFU but between MLCFU and lymphocytic leukaemia colony-forming units (unpublished observation). The existence of an advanced leukaemic stage, corresponding to an over-growth phase of myeloid leukaemia cell population is possibly comparable to the advanced situation in the human disease. In spite of its short duration in mice, its closer study, especially regarding changing growth fraction, will require further investigation.

### REFERENCES

Axelrad, A. A. and Steeves, R. A.—(1964) *Virology*, **24**, 513.

Bergsagel, D. E. and Valeriote, F. A.—(1968) *Cancer Res.*, **28**, 2187.

Bruce, W. R. and van der Gaag, H.—(1963) *Nature, Lond.*, **199**, 79.

Bruce, W. R. and Meeker, B. E.—(1964) *J. natn. Cancer Inst.*, **32**, 1145.

Bruce, W. R., Meeker, B. E. and Valeriote, F. A.—(1966) *J. natn. Cancer Inst.*, **37**, 233.

Kretchmar, A. L. and Conover, W. R.—(1968) *Proc. Soc. exp. Biol. Med.*, **129**, 218.

Lajtha, L. G. and Gilbert, C. W.—(1967) *Adv. biol. med. Phys.*, **11**, 1.

McCulloch, E. A. and Till, J. E.—(1964) *Radiat. Res.*, **22**, 383.

Odaka, T. and Yamamoto, T.—(1965) *Jap. J. exp. Med.*, **35**, 311.

Pluznik, D. H. and Sachs, L.—(1964) *J. natn. Cancer Inst.*, **33**, 535.

Pluznik, D. H., Sachs, L. and Resnitzky, P.—(1966) *Natn. Cancer Inst. Monogr.*, **22**, 3.

Siminovitch, L., McCulloch, E. A. and Till, J. E.—(1963) *J. cell. comp. Physiol.*, **62**, 327.

Skipper, H. E., Schabel, F. M., Jr. and Wilcox, W. S.—(1964) *Cancer Chemother. Rep.*, **35**, 3.

Steeves, R. A.—(1968) *Cancer Res.*, **28**, 338.

Steeves, R. A., Mirand, E. A. and Price, F. W.—(1968) *Cancer Chemother. Rep.*, **52**, 557.

Tanaka, T. and Lajtha, L. G.—(1969) *Br. J. Cancer*, **23**, 197.

Till, J. E. and McCulloch, E. A.—(1961) *Radiat. Res.*, **14**, 213.

Vadlamudi, S., Waravdekar, V. S., Choudry, J. N. and Goldin, A.—(1968) *Cancer Res.*, **28**, 1242.

Valeriote, F. A., Bruce, W. R. and Meeker, B. E.—(1968) *J. natn. Cancer Inst.*, **40**, 935.

Wodinsky, I., Swiniarski, J. and Kensler, C. J.—(1967*a*) *Cancer Chemother. Rep.*, **51**, 415.—(1967*b*) *Cancer Chemother. Rep.*, **51**, 423.